# Poor medical care for people with migraine in Europe – evidence from the Eurolight study

**DOI:** 10.1186/s10194-018-0839-1

**Published:** 2018-02-01

**Authors:** Zaza Katsarava, Maka Mania, Christian Lampl, Johanna Herberhold, Timothy J. Steiner

**Affiliations:** 1 Evangelical Hospital Unna, University of Duisburg-Essen, Essen, Germany; 2 Aversi Hospital, Tbilisi, Georgia; 3 Headache Medical Center, Department of Neurogeriatric Medicine and Remobilisation, Hospital of the Sisters of Charity, Linz, Austria; 4 Medical Faculty, Stradins University, Riga, Latvia; 5 Department of Neuromedicine and Movement Science, Faculty of Medicine and Health Sciences, NTNU Norwegian University of Science and Technology, Trondheim, Norway; 6 Division of Brain Sciences, Imperial College London, London, UK

**Keywords:** Headache, Migraine, Impact, Health care, Health policy, Europe, Eurolight project, Global campaign against headache

## Abstract

**Background:**

Migraine is prevalent everywhere, and disabling. It is also neglected: consequently, it is under-diagnosed and undertreated. We analysed data from the Eurolight study on consultations and utilization of migraine-specific medications as indicators of adequacy of medical care in Europe.

**Methods:**

Eurolight was a cross-sectional questionnaire-based survey in 10 European countries. Sampling was population-based in six (Germany, Italy, Lithuania, Luxembourg, Netherlands, Spain) and from consecutive patients attending general practitioners (GPs) for any reason in three (Austria, France, UK). Additional samples in Netherlands and Spain, and the only sample from Ireland, were recruited by lay headache organisations. We recorded migraine prevalence and frequency, and utilization of medical services and medications (acute and preventative).

**Results:**

Among 9247 participants (mean age 43.9 ± 13.9 years, M/F ratio 1:1.4), 3466 (37.6%) were diagnosed with migraine (definite or probable). Of these, 1175 (33.8%) reported frequent migraine (> 5 days/month) and might clearly expect benefit from, and therefore had need of, preventative medication. In population-based samples, minorities of participants with migraine had seen a GP (9.5–18.0%) or specialist (3.1–15.0%), and smaller minorities received adequate treatment: triptans 3.4–11.0%, with Spain outlying at 22.4%; preventative medication (1.6–6.4% of those eligible, with Spain again outlying at 13.7%). Proportions were greater in GP-based samples (13.6–24.5% using triptans, 4.4–9.1% on preventative medication) and among those from lay organisations (46.2–68.2% and 16.0–41.7%). Participants with migraine who had consulted specialists (3.1–33.8%) were receiving the best care by these indicators; those treated by GPs (9.5–29.6%) fared less well, and those dependent on self-medication (48.0–84.2%) were, apparently, inadequately treated.

**Conclusion:**

In wealthy European countries, too few people with migraine consult physicians, with proportionately too many of these seeing specialists, and migraine-specific medications are used inadequately even among those who do. These findings represent yet another call for action in Europe to improve care for people with headache. Education of both health-care providers and the public should be central to this action.

## Background

Headache disorders, including migraine, are extremely common [1]. From a public-health perspective, they are also among the most disabling at population level: according to the Global Burden of Disease (GBD) study, headache disorders collectively are the third highest cause in the world of years of healthy life lost to disability (YLDs), migraine alone being sixth (third in those aged under 50 years) [2–5]. The lost-productivity and consequential financial costs are enormous [6].

It might be expected that headache disorders would, everywhere, be considered important: as a personal medical problem by people directly affected by them, and as a public-health priority by health-care providers and health policy-makers. The reality is different. People with headache are under-diagnosed and undertreated not only in poorer countries with limited resources and restricted access to medical care but also, evidence indicates, in wealthy Europe and North America [7, 8]. We sought to verify this in Europe by analysing data from the Eurolight study.

Eurolight was an initiative supported by the European Commission Executive Agency for Health and Consumers (EHAC), and a partnership activity within the Global Campaign against Headache [9, 10] conducted by *Lifting The Burden* (LTB), a UK-registered non-governmental organization in official relations with the World Health Organization [11]. Eurolight gathered data on headache disorders in a cross-sectional survey in 10 countries, which together represented > 60% of the adult population (18–65 years) of the European Union (EU): Austria, France, Germany, Ireland, Italy, Lithuania, Luxembourg, Netherlands, Spain and United Kingdom (UK) [12]. The survey included diagnostic enquiry regarding primary headaches and further enquiry into headache-attributed burden and utilisation of medical services and medication for headache. Here we present data on consultations and use of migraine-specific acute and preventative medications, and analyse these as indicators of adequacy of medical care for people with migraine in Europe.

## Methods

### Ethics

The National Ethics Committee of Luxembourg gave overall approval of the protocol and provisions for data protection. Further approvals were obtained from national and/or local ethics committees wherever needed, since the methods for recruitment of participants differed between countries. In every country, prospective participants received written information explaining the project and its purpose.

### Study design

The methods of the Eurolight project have been described in detail elsewhere [12], and are summarised here.

Eurolight was a cross-sectional questionnaire-based survey of adults (18–65 years) in the EU, sampling from 10 countries. The sampling and questionnaire-distribution methods, summarised in Table 1, varied between countries according to what was feasible [12]: in six countries (Germany, Italy, Lithuania, Luxembourg, Netherlands, Spain), samples were population-based; in three (Austria, France, UK), general practitioners (GPs) recruited consecutive patients attending for any reason. Additional samples in Spain and Netherlands, and the only sample in Ireland, were recruited through lay organisations [12].

**Table 1:** Summary of sampling and data collection methods in each country [adapted from reference [12]

Country	Denominator (n)	Responders (n)	Responder proportion (%)	Gender (% female)	Target population and mode of distribution of questionnaire
Studies with a general-population basis					
Germany	3000	338	11.3	57	Random general-population sample from urban and rural areas, contacted by regular post
Italy	3500	500	14.3	58	Stratified general-population sample from urban and rural areas, contacted by regular post
Lithuania	1137	616	54.2	59	General-population sample in and around Kaunas (urban and rural), contacted by door-to-door cold-calling and personally interviewed by trained medical students
Luxembourg	6498	2023	31.1	58	Stratified general-population sample contacted by regular post
Netherlands-population	unknown	2414	incalculable	50	Stratified general-population sample contacted by internet
Spain-workplace	1700	999	58.8	59	Stratified sample of postal services employees, contacted by internal post by occupational health physicians
Studies conducted in general practice settings					
Austria	unknown, but not > 6000	646	incalculable	70	Consecutive patients consulting GPs or neurologists for any reason; questionnaire handed directly
France	2400	876	36.5	68	Consecutive patients consulting GPs for any reason; questionnaire handed directly
UK	720	128	17.8*	65	Consecutive patients attending GPs for any reason; questionnaire handed directly
Studies among members of lay headache organizations					
Ireland	members 1500 relatives unknown	195 73	13.0 incalculable	66	Members of Migraine Association of Ireland and their non-biological relatives, contacted by regular post
Netherlands-lay	members 500 partners unknown	337 115	67.4 incalculable	57	Random sample of members of Nederlandse Vereniging van Hoofdpijnpatiënten and (where existing) their non-headache-affected partners, contacted by regular post
Spain-lay	300	272	90.7	62	Members of Asociacion Española de Pacientes con Cefalea (AEPAC) and their family; questionnaires distributed by hand via helpers of AEPAC

The survey used the same structured questionnaire in all countries [13]. It had multiple parts. Demographic enquiry included age, gender, marital status and socio-economic status. Screening questions for headache (with an enquiry timeframe of the preceding year) were followed, in those responding positively, by headache-diagnostic questions based on ICHD-II [14]. To avoid diagnostic confusion, participants identifying more than one headache type were asked to report only on the one that was most bothersome to them. Enquiry into health-care utilisation entailed questions on use for headache of acute and preventative medications, consultations for headache (yes or no) with nurse, GP, neurologist or headache specialist, investigations for headache (MRI, CT, X-rays of the neck, blood tests, ophthalmic examination), and admissions (number) to hospital because of headache.

### Analysis and statistics

Diagnoses were made from questionnaire responses by computerized algorithm [15]. This first identified, and separated, participants reporting headache on ≥15 days/month, of whom additional questions had enquired into frequency of acute medication use. Probable medication-overuse headache (pMOH) was diagnosed when, in addition, simple analgesics were used on ≥15 days/month or medication including compound analgesics, opioids, triptans and/or ergots was taken on ≥10 days/month. A diagnosis of pMOH trumped all other diagnoses. The remainder of this group were diagnosed as “other headache on ≥15 days/month”. To all others, who had headache on < 15 days/month, the algorithm applied ICHD-II criteria for definite migraine, definite tension-type headache (TTH), probable migraine and probable TTH in that order.

Analysis focused on participants in whom migraine was diagnosed, not distinguishing between those meeting criteria for definite migraine or probable migraine [12, 15, 16]. We identified those with migraine on > 5 days/month as clearly eligible for preventative medication. We selected the following as indicators of adequacy of care: a) proportion receiving migraine-specific acute medications (triptans); b) proportion of those clearly eligible receiving any preventative medication; c) proportions receiving medical care through GP or specialist (neurologist or specialist in headache medicine). In our analysis, consultation with specialist(s) trumped consultation with GP(s).

Statistical analyses were performed using SPSS. We describe categorical variables in terms of frequency (n) and proportions (%), and continuous variables in terms of means ± standard deviations (SDs). We used 2 × 2 chi-squared to compare proportions receiving treatments in specialist and primary care.

## Results

Eurolight collected 9247 correctly completed questionnaires from participants in the 10 countries (mean age 43.9 ± 13.9 years, M/F ratio 1:1.4 [Table 1]). The principal results have been reported in detail previously [17]. Here we analysed the data of 3466 people (37.6%) with migraine (definite or probable) (Table 2).

**Table 2:** Sociodemographic features of participants with migraine (*N* = 3466)

Country	n	Females (%)	Age (years) (mean ± SD)	Living with spouse or partner (%)	Employed or self-employed (%)
Studies with a general-population basis					
Germany	109	67.9	41.6 ± 11.9	57	65
Italy	221	70.1	40.2 ± 11.7	94	71
Lithuania	149	80.5	42.5 ± 12.3	67	66
Luxembourg	669	67.9	38.2 ± 11.2	73	69
Netherlands-population	815	60.2	40.5 ± 12.5	68	69
Spain-workplace	401	68.6	41.7 ± 11.1	71	83
Studies conducted in health-care settings					
Austria	263	81.4	44.2 ± 13.5	74	67
France	337	76.0	43.1 ± 13.5	78	64
UK	49	100.0%	41.7 ± 16.0	64	63
Studies among members of lay headache organizations					
Ireland	152	88.8	48.5 ± 13.1	71	59
Netherlands-lay	195	83.1	46.5 ± 10.9	78	65
Spain-lay	106	76.4	40.8 ± 11.0	71	84

The demography of participants with migraine was broadly similar in all countries: they were on average approximately 40 years old, two thirds or more were female, most were married or living with a partner, and most were employed or self-employed (Table 2). Across countries, one third (1175; 33.8%) reported frequent migraine (> 5 days/month) (Table 3) and therefore had clear need of preventative medication.

**Table 3:** Utilization of medical care by participants with migraine (N = 3466)

Country	N	Using triptans (of all with migraine) n (%)	Migraine on ≥5 days/month n (%)	Using preventative medication (of those with migraine on ≥5 days/month) n (%)	Consulting health professionals n (%)			
					Specialist	General practitioner	Non-medical	None
Studies with a general-population basis								
Germany	109	12 (11.0)	42 (38.5)	1 (2.4)	7 (6.4)	14 (12.8)	5 (4.6)	83 (76.1)
Italy	221	14 (6.3)	61 (27.6)	1 (1.6)	14 (6.3)	21 (9.5)	15 (6.8)	171 (77.4)
Lithuania	149	5 (3.4)	62 (41.6)	2 (3.2)	16 (10.7)	23 (15.4)	2 (1.3)	108 (72.5)
Luxemburg	669	48 (7.2)	219 (32.7)	10 (4.6)	39 (5.8)	105 (15.7)	27 (4.0)	498 (74.4)
Netherlands-population	815	75 (9.2)	171 (20.8)	11 (6.4)	25 (3.1)	106 (13.0)	36 (4.4)	648 (79.5)
Spain-workplace	401	90 (22.4)	153 (38.2)	21 (13.7)	60 (15.0)	72 (18.0)	28 (7.0)	241 (60.1)
Studies conducted in general practice settings								
Austria	263	37 (14.1)	106 (40.3)	7 (6.6)	46 (17.5)	26 (10.0)	14 (5.3)	177 (67.3)
France	337	46 (13.6)	91 (27.0)	4 (4.4)	7 (2.1)	81 (24.0)	22 (6.5)	227 (67.3)
UK	49	12 (24.5)	22 (44.9)	2 (9.1)	11 (22.4)	7 (14.3)	0 (0)	31 (63.3)
Studies among members of lay headache organizations								
Ireland	152	94 (61.8)	78 (51.3)	23 (29.5)	34 (22.4)	45 (29.6)	6 (3.9)	67 (44.1)
Netherlands-lay	195	133 (68.2)	120 (61.5)	50 (41.7)	66 (33.8)	26 (13.3)	21 (11.0)	82 (42.1)
Spain-lay	106	49 (46.2)	50 (47.2)	8 (16.0)	25 (23.6)	27 (25.5)	10 (9.4)	44 (41.5)

Utilisation of migraine-specific medications varied greatly between countries. The ranges were, for triptans, 3.4–68.2% of all participants with migraine and, for preventative medications, 1.6–41.7% of those deemed clearly eligible for them. These ranges were considerably distorted by the studies among members of lay headache organisations. In population-based samples, 3.4–22.4% of participants with migraine used triptans and 1.6–13.7% of those eligible used preventative medication. In this group, Spain-workplace was an outlier, with values in each case double those of the next highest, while Lithuania appeared to be an outlier for low use of triptans (3.4%) and Italy for low use of preventative medication (1.6% of those eligible) (Table 3). Proportions were higher in GP-based samples (13.6–24.5% using triptans, 4.4–9.1% on preventative medication), and much higher among those from lay organisations (46.2–68.2% and 16.0–41.7%) (Table 3).

Proportions of people receiving specific acute or preventative medications did not depend an age or socio-economic or marital status (data not shown).

**Fig. 1:**
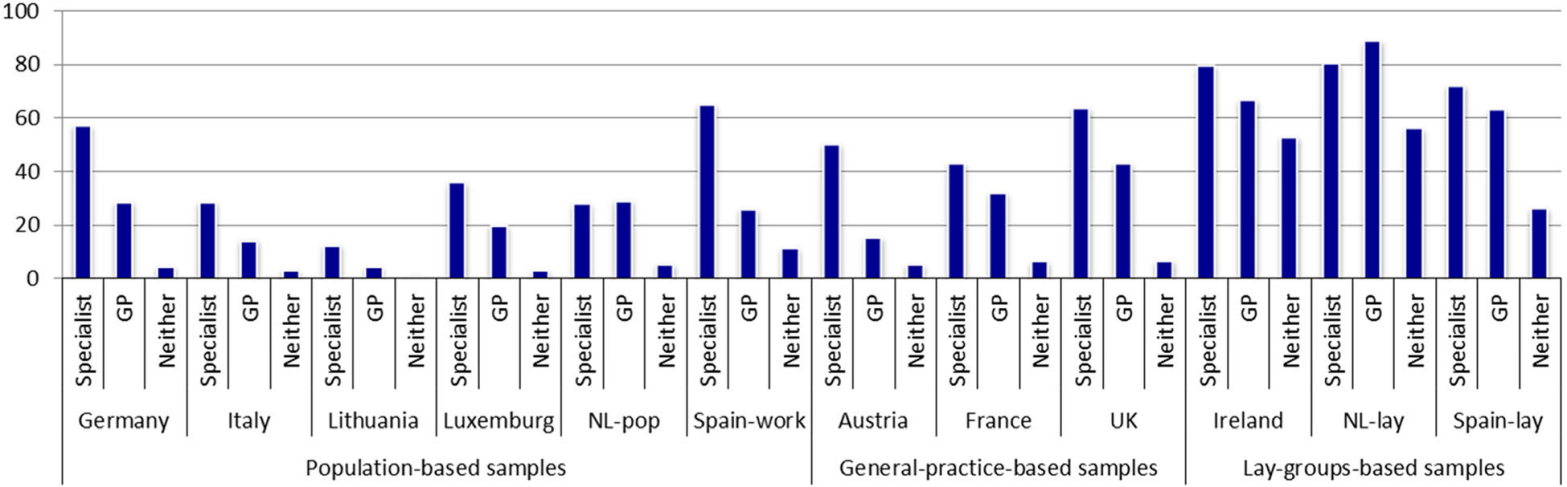
Proportion (%) of participants with migraine using triptans, by country and by whom consulting. NL-pop: Netherlands-population; Spain-work: Spain-workplace; NL-lay: Netherlands-lay

**Fig. 2:**
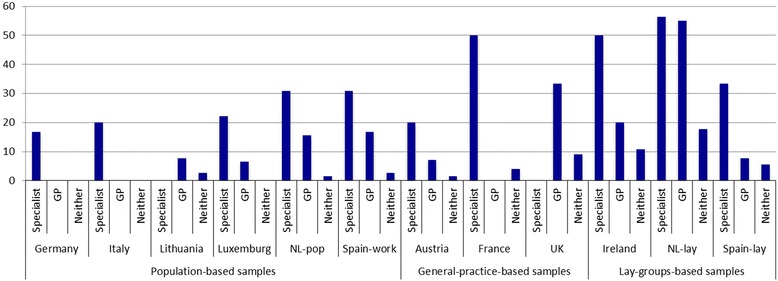
Proportion (%) of eligible participants with using migraine-preventative treatment, by country and by whom consulting. NL-pop: Netherlands-population; Spain-work: Spain-workplace; NL-lay: Netherlands-lay

These medications were not widely available without prescription; therefore, they were accessible only to a limited extent by people treating themselves or consulting a nurse rather than a physician. With regard to triptan use, not only was this highly dependent on consultation with a physician but also the proportions using triptans were greater in those seeing specialists (mean 51.3%) than in those treated by GPs (mean 35.9%; chi-squared = 62.1; *p* < 0.001) (Fig. 1). Similarly, those clearly eligible to receive preventative medications were more likely to do so from specialists (mean 26.0%) than from GPs (mean 14.1%; chi-squared = 10.1; p < 0.001) (Fig. 2). Participants with migraine who had consulted specialists (2.1–33.8% across all studies) were receiving the best care by these indicators; those treated by GPs (9.5–29.6%) fared less well, and the larger numbers dependent on self-medication (48.0–84.2%) appeared to be inadequately treated. Probability of consultation was itself highly dependent on the source of the sample (Table 3). In the studies with a general-population basis, a minority of participants (15.8–33.0%) had done so: 3.1–15.0% had consulted a specialist (averaged across countries: 6.8%), and 9.5–18.0% had seen a GP (average: 14.4%). In studies conducted in general practice settings, 26.1–36.7% of participants had consulted, and in studies among members of lay headache organizations, 47.1–52.0% had done so, with the ranges for specialist and GP as shown in Table 3. The differences reflect bias in the latter samples.

## Discussion

EU countries represent a relatively wealthy area of the world, albeit with some variation. Our study in 10 of these countries confirms that, even among these, migraine is under-treated. At best in the general-population samples (setting aside Spain-workplace), 26.1% of those with migraine in Lithuania were consulting a physician for it, triptans were in use by 11.0% in Germany, and preventative medication by 6.4%, of those who appeared to be clear candidates for it, in Netherlands. At worst, the proportions were as low as 15.8% consulting (in Italy), 3.4% using triptans (in Lithuania) and 1.6% of those eligible using preventative medication (again in Italy). The Spanish workplace-based sample fared better on all counts – 33.0%, 20.4% and 13.7%, suggesting facilitated access to health care among this sample of employed people. Contact with GPs, and with specialists more so, was associated with higher use of these medications, which is unsurprising not only because they are in the main prescription-only but also because worse-affected people might be more likely to consult.

The samples recruited by general practitioners, and more so those from lay organisations, showed higher proportions consulting and utilising specific medications, which we attribute to the expected selection bias in these two groups. The latter did better for use of triptans (60.9% of those with migraine), but, even among these, fewer than one third (32.7%) of those clearly eligible were using preventative drugs.

While the proportions in contact with doctors and receiving migraine-specific drugs in this study were self-evidently low, at issue here is what, in a perfect world, they should be. In a resource-limited world, not everyone with migraine can receive professional health care, but neither should they: people with relatively mild and infrequent attacks who self-manage adequately with over-the-counter (OTC) remedies should not be brought into the health-care system. Published recommendations are that about 50% of people with migraine can adequately self-manage [18], suggesting that the other 50% need to consult at some level. To a large extent, need for consultation is driven by the inefficacy of the OTC medications and the need for prescription drugs. Assessed by the outcome measure *sustained headache relief* (SHR), arguably an adequate if imperfect outcome [19], aspirin 900–1000 mg has a predicted efficacy of 39% (headache relief at 2 h [HR] 52%) [20] and ibuprofen 400 mg of 45% (HR 57%) [21]. For paracetamol 1000 mg there are no data for SHR but HR = 56% [22].

What our findings indicate is that, even in these relatively well-resourced countries, perhaps only half of those who would be expected to benefit from physician-consultation actually received it. Of those who did, proportions seen by GPs (9.5–18.0% in the population-based groups) and specialists (3.1–15.0%) were not very dissimilar. It is true that those seeing specialists might also be seeing GPs since, in such cases, we recorded only the former. But the question raised is this: is it necessary, or cost-effective, for migraine care to be thus distributed between specialists and GPs? Recommendations are that only the small minority of complicated cases should be referred from primary care to specialists [7, 18].

Furthermore, the quality of that care, when it was received, appeared far from perfect. If need for triptans is a reason to consult physicians, at least the majority of those doing so might be expected to receive them. While this was barely achieved (51.3%) among those seeing specialists, only 35.9% did so from GPs. Worse, while use of preventative medication by people with > 5 migraine days/month ought by any objective standard to be close to 100%, the best we saw outside the self-selecting lay-organization members was 13.7%, and this was in an employee group with, probably, facilitated access to care.

The picture is therefore not encouraging: as reflections of reach and adequacy of headache services for headache, these findings indicate depressingly poor performance in the EU. The situation may even be worse than suggested. On the subject of bias, the Eurolight project as a whole suffered from low participation proportions – on average, 27.5% in the non-lay samples [17]. Some participation bias was likely, with the probability that, through self-selection, samples were preferentially constituted of more demanding participants, whose needs and health-care utilisation were likely to be higher than average.

Several earlier studies have found much the same. In Germany, among 7431 adults, awareness of migraine was low among those who had it, as was recognition of it by health-care providers [23]. Also in Germany, in three regions of the country, a population-based study of 10,000 people found only 8% of those with migraine used triptans and only 2.3% received preventative treatment, both positively associated with socio-economic status [24] and suggesting inequitable access to health care. Similar results emerged from France: among approximately 10,000 people studied, about 60% of the 1179 with migraine were unaware of the diagnosis, only 20% used triptans and only 2.3% received preventative medication [25]. In Italy, a study of 2675 patients in 10 headache centres revealed that only 26.8% with migraine were previously correctly diagnosed, only 17.2% were using triptans and only 4.8% were using migraine-specific preventative medications [26]. In Sweden, a recent cross-sectional study in Stockholm analysed data from a pharmaceutical registry. It found that, of patients with a diagnosis of migraine and a high attack frequency (self-reported), and utilizing triptans for acute treatment, only 4% received preventative medication [27].

Some limitations of the study should be considered. First, Eurolight recruited from 10 EU countries with diverse sampling methods: some samples were population-based, others were general-practice-based and some were recruited through lay organisations [12]. A degree of interest-bias was very likely [17]. If this led to recruitment of more seriously affected patients, arguably our findings of under-treatment appear in even worse light. There was consistency in these findings, with more adequate medication among responders from lay groups, who were likely to be more vocal in their demands. Patients seeing specialists received better care than those treated by GPs, but important is that medical care was insufficient in *all* groups. A second limitation was that the cross-sectional Eurolight survey, while observing variations between countries, could not enquire into the reasons for under-treatment [12] (which might include differences in costs, health-seeking behaviour, culture and tradition, and structure and accessibility of health-care systems).

The last 10 years have been crucial in improving knowledge of the prevalence and impact of migraine, beginning in 2007 with a systemic review of the existing literature [28]. The gaps in knowledge revealed by this review have been progressively filled by population-based studies undertaken by the Global Campaign against Headache [9, 10, 29]. The GBD studies have ranked migraine as the sixth highest cause of disability worldwide, third in both men and women aged under 50 years [4, 5]. The Eurolight study found that nearly one fifth of males and over a quarter of females with migraine reported the loss of > 10% of productive days [17]. It also demonstrated that the burden of migraine was not confined to attacks: there was measurable interictal burden also [30]. The estimated financial costs to the EU are huge [6]. Migraine remains to a very large extent to be untreated despite all this, and despite that migraine is a very treatable disorder [7, 31, 32]. This is a failure of health care with major adverse health and economic consequences.

While some access to a wider range of drugs is achieved by consulting GPs, and rather more by seeing headache specialists, this is emphatically not a call for everyone with migraine to see specialists. Rather, it is a plea to curtail the insouciance to which migraine appears condemned [8]. We identify four needs, largely to be met by education at multiple levels.

First, people with migraine should learn, through public health-education programmes, that migraine is a neurobiological disease that can often be effectively treated with correct usage of OTC drugs [7, 31, 32]. Here, pharmacists can help, but otherwise people with migraine should consult a GP. Second, in order to relieve an otherwise insupportable load on specialists, health-care providers in general and GPs in particular need better knowledge of how to recognise, diagnose and treat migraine (along with the small number of other headache disorders that are of public-health importance) [18, 32]. Without this, the potential benefits of consulting GPs will be frustrated. This better knowledge will improve usage of available treatments, produce better outcomes, avoid wastage [33] and, importantly, reduce overall costs [7]. Third, headache services need to be structured, so that they might be delivered countrywide, efficiently and equitably to the very large number of people who stand to benefit from them [7, 18]. And fourth, and most important, is the urgent need for political recognition not only that the problem exists but also that it demands remedial action [3, 5, 7].

The alternative is that large numbers of people will remain without diagnosis or best treatment, with not much hope for change, their unmitigated personal disability burdens translating into lost productivity with reduced societal output reflected in gross domestic product. The drive to produce new drugs and devices for headache [34, 35] offers little apparent utility if, when developed, they will not reach most patients [36]. The choice between these alternatives – invest in headache services, or do nothing – is, surely, not a difficult decision.

## Conclusion

In wealthy European countries, too few people with migraine consult physicians, with proportionately too many of these seeing specialists, and migraine-specific medications are used inadequately even among those who do. These findings represent yet another call for action in Europe to improve care for people with headache. Education of both health-care providers and the public should be central to this action.
